# Bibliographical Department

**Published:** 1881-07

**Authors:** 


### BIBLIOGRAPHICAL DEPARTMENT.

"Nothing extenuate, nor set down aught in malice."

We are indebted to the courtesy of Prof. Garretson, Dean of the Philadelphia Dental College and Hospital of Oral Surgery, for the announcement—session 1881–2—circular and of the college, and the syllabi of the several chairs or branches taught in the institution. The name of Dr. Garretson is in itself a tower of strength, and all the professors seem to be men of ability.

*Quarterly Epitome of Practical Medicine and Surgery, being an American Supplement to Braithwaite's Retrospect. Part* 5. *March,* 1881. *W. A. Townsend, Publisher, New York.* $2.50 *a year, in advance.*

The above quarterly has just been received in exchange for the first time. The number before us is quite well filled with abstracts from many of the journals published in this country. No quotations or extracts appear from several well-known American journals, in the issue under notice, but those omissions may have been accidental, or owing to want of space.

A journal claiming to be an epitome of the progress of American medicine, should have all our journals at its command from which to draw its supplies, as neither partiality nor favoritism would be likely to prove advantageous to such an enterprise.

We cheerfully enroll its name on our list of exchanges.

*The Indiana Medical Reporter, a Monthly Journal of Medicine and Surgery. Edited by Drs. A. M. Owen, J. W. Compton, J. E. Har-*

*per, Arch. Dixon and J. Gardner*, has also been received since our last issue and placed among our exchanges ; and is an interesting journal. Published by Dr. J. W. Compton, at Evansville, Ind., and Chicago, Illinois.

THE PATHOLOGIST, edited by E. S. Bunker, M. D., New York City, was accidentally overlooked in our notices for May last. We wish it success.

*Chest Drainage in Empyema*, with cases read before the Iowa State Medical Society by George M. Staples, A. M., M. D., of Dubuque, Iowa, has been received from the author. His cases are interesting and well related.

*Shortening of Limbs after Fracture, By Louis A. Sayre, M. D.*, is to hand. It is a criticism on certain statements by Prof. Frank H. Hamilton, in the sixth edition of his work on "Fractures and Dislocations." It is much to be regretted that those prominent gentlemen should be so far at variance on the important subject at the head of this notice.

The Fourteenth Annual Announcement of the Boston Dental College, 1881–82, is an appreciated favor from the Dean—Prof. J. A. Follett. We note a new feature in *dental* education, viz : Two courses of lectures, one for junior students, and the other for seniors, thus making the college pupilage progressive over two years.

We are the happy recipients of the transactions of the *Ohio State Dental Society*—15th annual meeting ; the *Iowa State Dental Society*—18th annual session ; and Proceedings of the *New Jersey State Dental Society*, 1878-9–80.

